# Comparison of STI-related consultations among ethnic groups in the Netherlands: an epidemiologic study using electronic records from general practices

**DOI:** 10.1186/s12875-015-0281-2

**Published:** 2015-06-18

**Authors:** Petra J. Woestenberg, Aloysia A. M. van Oeffelen, Irina Stirbu-Wagner, Birgit H. B. van Benthem, Jan E. A. M. van Bergen, Ingrid V. F. van den Broek

**Affiliations:** Centre for Infectious Disease Control, National Institute for Public Health and the Environment (RIVM), Bilthoven, The Netherlands; Netherlands Institute for Health Services Research (NIVEL), Utrecht, The Netherlands; STI AIDS Netherlands, Amsterdam, The Netherlands; Department of General Practice, Academic Medical Center, Amsterdam, The Netherlands

**Keywords:** Sexually transmitted infections (STI), STD, HIV, Testing, Ethnicity, General practice, Primary care

## Abstract

**Background:**

Currently, surveillance of sexually transmitted infections (STIs) among ethnic minorities (EM) in the Netherlands is mainly performed using data from STI centers, while the general practitioner (GP) is the most important STI care provider. We determined the frequency of STI-related episodes at the general practice among EM, and compared this with the native Dutch population.

**Methods:**

Electronic medical records from 15–to 60-year-old patients registered in a general practice network from 2002 to 2011 were linked to the population registry, to obtain (parental) country of birth. Using diagnoses and prescription codes, we investigated the number of STI-related episodes per 100,000 patient years by ethnicity. Logistic regression analyses (crude and adjusted for gender, age, and degree of urbanization) were performed for 2011 to investigate differences between EM and native Dutch.

**Results:**

The reporting rate of STI-related episodes increased from 2004 to 2011 among all ethnic groups, and was higher among EM than among native Dutch, except for Turkish EM. After adjustment for gender, age, and degree of urbanization, the reporting rate in 2011 was higher among Surinamese [Odds Ratio (OR) 1.99, 95 % confidence interval (CI) 1.70-2.33], Antillean/Aruban (OR 2.48, 95 % CI 2.04-3.01), and Western EM (OR 1.24, 95 % CI 1.11-1.39) compared with native Dutch, whereas it was lower among Turkish EM (OR 0.48, 95 % CI 0.37-0.61). Women consulted the GP relatively more frequently regarding STIs than men, except for Turkish and Moroccan women.

**Conclusions:**

Most EM consult their GP more often for STI care than native Dutch. However, it remains unclear whether this covers the need of EM groups at higher STI risk. As a first point of contact for care, GPs can play an important role in reaching EM for (proactive) STI/HIV testing.

**Electronic supplementary material:**

The online version of this article (doi:10.1186/s12875-015-0281-2) contains supplementary material, which is available to authorized users.

## Background

In the Netherlands, sexually transmitted infection (STI) testing and care is mainly provided by general practitioners (GPs) and specialized STI centers [1–3]. In general, GPs are the core primary care providers in the Netherlands and function as a ‘gatekeeper’ to secondary health care. Nearly all Dutch inhabitants are registered at a general practice. Specialized STI centers provide additional low-threshold and free of charge STI care to certain high-risk groups [2, 4]. It is estimated that the annual number of STI-related consultation at the GP is over 300,000, while there were about 120,000 consultations at the STI clinic in 2012 [2].

An important high-risk group eligible for free of charge STI testing at the STI centers are people originating from STI/HIV endemic areas (Turkey, Latin America (like Surinam, the Netherlands Antilles), Africa (like Morocco), eastern Europe, Asia) [2, 4]. As in other countries, certain ethnic minorities (EM) are at higher risk to acquire STIs than the majority population, which is related to a higher STI prevalence in their country of origin, sexual networking or higher sexual risk behavior [5–10]. At the STI center, but also in other settings, people with a non-Dutch ethnicity are more often diagnosed with an STI than native Dutch. For example, in 2013 20.1 % of the Surinam/Antillean/Aruban EM, 18.3 % of the Sub-Saharan African EM and 16.1 % of the Moroccan/Turkish EM were diagnosed with an STI at the STI center compared to 13.9 % of the native Dutch population. Also in a population-based study, the chlamydia positivity rate was higher among Surinam/Antillean EM (8.2 %) and Moroccan/Turkish EM (3.1 %) than among native Dutch (1.8 %) [2, 11–16]. Therefore, it is important that EM are properly tested for STIs. This may not only reduce the risk of complications and improve the prognosis for the individual, but may also reduce transmission of STIs in the population.

Because the patients’ ethnic background is not registered at the general practice, surveillance of STIs and STI testing among EM in the Netherlands is mainly performed using data from STI centers. However, the majority of the STIs is diagnosed at the GP, and most people with signs and symptoms suggestive of an STI consult their GP and not an STI center [3, 17, 18]. Although EM (originating from STI/HIV endemic areas) are indicated as high-risk groups for whom opportunistic STI-testing is recommended, it is currently unknown if these groups consult the GP for STI-related issues as frequently as native Dutch [19]. There are indications that EM consult the GP less frequently [20]. General practice surveillance data can give a broader insight in the frequency of STI consultations among EM in the general population and improve the surveillance of STIs [3, 21].

In the Dutch population registry, ethnic background of inhabitants is recorded by means of (parental) country of birth. By matching general practice data with data from the population registry, it became possible to determine the patients’ ethnicity. Although a distinction based on ethnic background may be perceived as stigmatizing, it does provide more insight into primary care use among EM and it enables a more focused approach regarding service provision [22]. In this paper, we answered the research question: what are the differences in the number of GP consultations for STI-related issues between EM and native Dutch in the period 2002-2011?

## Methods

### Data sources

We used the Netherlands Institute for Health Sciences Research (NIVEL) Primary Care Database (NIVEL-PCD) to obtain the electronic medical records of participating general practices from 2002 to 2011. The number of general practices included in the NIVEL-PCD is dynamic and fluctuates from year to year (about 70 to 120 practices per year), but its composition is guarded so that the practices are representative of all Dutch general practices with regard to geographical distribution and degree of urbanization. The patients registered in these participating practices are also representative for the Dutch population with respect to gender and age [23].

In the NIVEL-PCD, GPs record information on the diagnosis and prescriptions of each consultation. Diagnoses are recorded using the International Classification of Primary Care 1 (ICPC-1) codes [24]. Prescriptions are recorded using Anatomical Therapeutic Chemical Classification System (ATC) codes [25].

The medical records were matched to individual records in the Dutch population registry using a unique anonymous patients’ identifier (based on gender, date of birth, and postal code). The degree of urbanization of the patients’ residence (five categories, based on population density per postal code area), obtained from Statistics Netherlands, was also linked to the database [26]. Both NIVEL and Statistics Netherlands, responsible for the management of the population registry, approved this study. Using the data in this manner and for this purpose is permitted under Dutch law and no additional ethical approval is needed.

### Study population

We included all 15–to 60-year-old patients registered at the participating GPs in the NIVEL-PCD during the study period 2002 to 2011 who could be identified in the population registry.

### STI-related episodes

For episode-based registration, multiple consultations concerning one health problem were grouped into disease episodes as registered by the GP or as constructed by the validated application EPICON, that groups consultations with similar ICPC-codes occurring less than two months apart [27]. An STI-related episode was defined as an episode with an ICPC code for a positive STI-diagnosis or an episode with the ICPC code for fear of STI or HIV/AIDS (Table 1). GPs register the latter codes in case of a consultation where STI-related questions were discussed and/or an STI test was done, but no STI was diagnosed. Because there is no ICPC code for chlamydia, we combined prescription data with specific ICPC codes to define a chlamydia episode (Table 1) [3, 21]. There were probably more episodes during which STI-related issues were discussed or STI-tests were performed, but we used only the final ICPC code assigned to each episode. Patients can have multiple STI-related episodes per year. Hepatitis B and C are not included in this study, since there are no specific ICPC-1 codes for these infections. There is a code for hepatitis in general, but this also includes other infections.

**Table 1:** Definition of STI-related episode

STI-related episode	Men	Women
	ICPC-1 code	ICPC-1 code
Fear for STI or HIV/AIDS		
Fear of STI	B25	B25
Fear of HIV/AIDS	Y25	X23
STI-diagnosis		
HIV infection/AIDS	B90	B90
Syphilis	Y70	X70
Gonorrhea	Y71	X71
Trichomoniasis	Not included	X73
Herpes genitalis	Y72	X90
Genital warts	Y76	X91
Non-specific urethritis	U72	Not included
Chlamydia^a^		
- Orchitis/Epididymitis	Y74	
- Other genital disease	Y99	
- Pelvic inflammatory disease		X74
- Vaginitis		X84
- Cervicitis		X85

^a^Chlamydia is defined by the ICPC-1 codes in combination with one of the following ATC prescription codes: J01FA10, J01AA02, J01FA01, J01MA01, J01MA02, J01CA04 (for women only)

### Ethnicity

**Fig 1:**
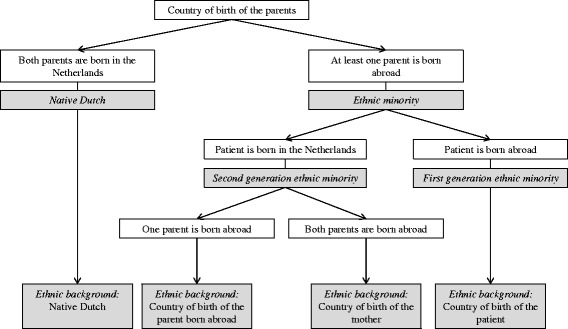
Flowchart to determine ethnic background

We used (parental) country of birth, as recorded in the population registry, to define the ethnicity of patients and the generation of EM (Fig. 1) [28]. Patients with at least one parent born abroad are considered EM. First-generation EM are themselves also born abroad, and second-generation EM are themselves born in the Netherlands. Patients with both parents born in the Netherlands are considered native Dutch. In this study, we focused on ethnicities that were most prevalent among the 15–to 60-year-old Dutch population in 2002 to 2011: native Dutch (79.3 %), Moroccan (1.9 %), Turkish (2.3 %), Surinamese (2.3 %), Antillean/Aruban (0.9 %). Other ethnicities were classified as Western (8.5 %) or non-Western (4.8 %) [29].

### Statistical analyses

Per ethnic group, we calculated the proportion of all registered episodes that were STI-related. In order to compare STI consultations at the GP between ethnic groups, the reporting rate of STI-related episodes was calculated per ethnic group as the number of STI-related episodes per 100,000 patient years (PYs). Gender differences were analyzed as well as trends over time using chi-square for trend.

We performed uni–and multivariable logistic regression analyses on the outcome STI-related episodes to investigate the difference between EM and native Dutch. Analyses were adjusted for gender, age and degree of urbanization. We performed the logistic regression analyses on the data of 2011 only, because this represents the most recent situation. A p-value less than 0.05 was considered statistically significant. Analyses were performed using SPSS version 20.

## Results

### Study population

The number of participating GPs in the NIVEL-PCD, and consequently the number of registered patients, fluctuated each year. The mean number of registered 15–to 60-year-old patients was 383,712 per year [range: 289,804–475,122] which is about 2.5 % of the total Dutch 15–to 60-year-old population [29].

Of all 15–to 60-year-old registered patients, 66.8 % could be identified in the population registry. Of the patients who consulted the GP, 88.4 % could be identified. Reasons for the inability to identify patients were for example an unknown date of birth or (recent) change of address. Identified patients were somewhat older; of the 45–to 60-year-old patients, 71.6 % could be identified compared with 61.3 % of the 25- to 34-year-old patients.

Of the study population 80.8 % was native Dutch, 1.7 % Moroccan, 2.0 % Turkish, 2.1 % Surinamese and 0.9 % was Antillean/Aruban. The majority of the EM were first-generation EM (59.3 %). Characteristics of the study population are presented in Additional file 1.

### STI-related episodes by ethnicity

Between 2002 and 2011 a total of 3,749,370 episodes were registered, of which 21,065 (0.6 %) were STI-related. The percentage of episodes registered as STI-related differed by ethnicity and generation of EM (Table 2). The highest percentage was observed among Antillean/Aruban EM; of all registered episodes 2.0 % was STI-related. The lowest percentages were observed among Turkish EM (0.4 %) and native Dutch (0.5 %).

**Table 2:** Number of episodes per patient year and the proportion of episodes that was STI-related. In the period 2002 to 2011, by ethnicity and generation of ethnic minorities (EM)

	Patient years	Number of episodes	Number of episodes per patient year [95 % CI]	Number of STI-related episodes	% STI-related episodes of all episodes [95 % CI]
Total	2,563,599	3,749,370	1.46 [1.46 – 1.46]	21,065	0.56 [0.55 – 0.57]
Ethnicity					
Native Dutch	2,071,677	2,970,801	1.43 [1.43 – 1.44]	14,976	0.50 [0.50 – 0.51]
Moroccan	42,965	76,737	1.79 [1.77 – 1.80]	524	0.68 [0.63 – 0.74]
Turkish	52,051	100,473	1.93 [1.92 – 1.94]	446	0.44 [0.40 – 0.49]
Surinamese	53,212	98,033	1.84 [1.83 – 1.85]	1,098	1.12 [1.06 – 1.19]
Antillean, Aruban	23,570	35,303	1.50 [1.48 – 1.51]	714	2.02 [1.88 – 2.17]
Non-western, other	97,887	149,218	1.52 [1.52 – 1.53]	1,233	0.83 [0.78 – 0.87]
Western, other	222,237	318,805	1.43 [1.43 – 1.44]	2,074	0.65 [0.62 – 0.68]
Generation of EM					
1^st^ generation	291,911	491,214	1.68 [1.68 – 1.69]	3,121	0.64 [0.61 – 0.66]
2^nd^ generation	200,011	287,355	1.44 [1.43 – 1.44]	2,968	1.03 [1.00 – 1.07]

**Fig 2:**
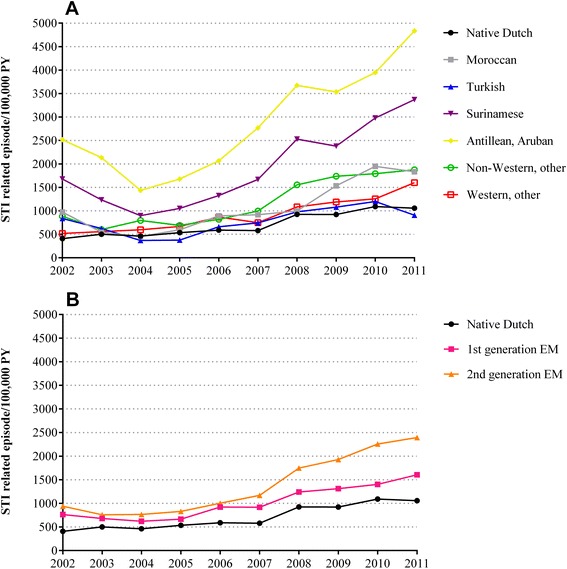
Reporting rate of STI-related episodes per year (2002 to 2011). **a**) Reporting rate of STI-related episodes by ethnicity. **b**) Reporting rate of STI-related episodes by generation of ethnic minorities (EM)

**Fig. 3:**
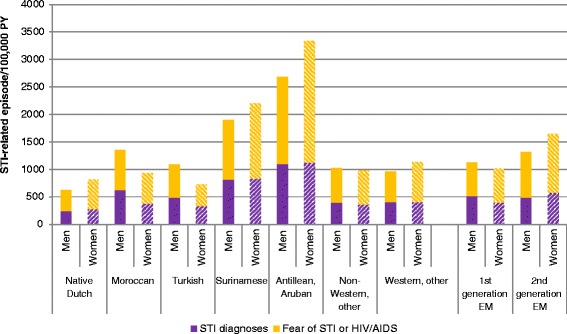
Reporting rate of STI-related episodes divided into STI diagnoses and fear of STI or HIV/AIDS. In the period 2002 to 2011, by ethnicity, generation of ethnic minorities (EM), and gender

The overall reporting rate of STI-related episodes at the GP increased from 479 per 100,000 PY in 2002 to 1,240 per 100,000 PY in 2011. Among some ethnic groups the reporting rate first declined from 2002 until 2004 followed by an increase (Fig. 2). The increasing trend from 2004 onwards was significant across all ethnic groups and generations of EM (p < 0.001). Antillean/Aruban and Surinamese EM had more often an STI-related episode at the GP than other ethnic groups during the entire study period (Fig. 2a) and second-generation EM more often than first-generation EM (Fig. 2b). Except for Moroccan, Turkish, other non-Western EM and first-generation EM, women had a higher reporting rate of STI-related episodes than men (Fig. 3).

The reporting rate of a positive STI-diagnosis was the highest among Antillean/Aruban and Surinamese EM, in line with the higher rate of STI-related episodes. The ratio of STI-diagnoses to all STI-related episodes differed between ethnic groups. Among Moroccan men and first-generation EM men, proportionally more positive STI-diagnoses were registered during STI-related episodes than among other ethnic groups, for example native Dutch and Antillean/Aruban women. In general, the episodes registered as STI-related less often included a positive STI-diagnosis among women than among men of the same ethnicity, except for Turkish EM (Fig. 3).

Univariable logistic regression analyses showed that, in 2011, all EM (both first and second generation) had more often an STI-related episode than native Dutch, except for Turkish EM in which results were similar to native Dutch (Table 3). Adjustment for gender did not affect these results. Further adjustment for age did attenuate but not remove the associations. Only for Turkish EM adjustment for age resulted in less STI-related episodes compared with native Dutch. After additional adjustment for degree of urbanization, there were no differences between Moroccan or other non-Western EM and native Dutch, and between first-generation EM and native Dutch. Differences in demographic characteristics between ethnic groups are presented in the Additional file 2.

**Table 3:** Crude and adjusted Odds Ratio for STI-related episodes by ethnicity and generation of ethnic minorities. Adjusted for gender, age and degree of urbanization, using data from 2011

		STI-related episode			
	n	Crude OR [95 % CI]	aOR [95 % CI]^a^	aOR [95 % CI]^b^	aOR [95 % CI]^c^
Ethnicity					
Native Dutch	221,184	Reference	Reference	Reference	Reference
Moroccan	6,220	**1.72 [1.41 – 2.09]**	**1.71 [1.40 - 2.08]**	**1.27 [1.04 - 1.55]**	0.92 [0.76 – 1.13]
Turkish	7,489	0.87 [0.68 – 1.11]	0.87 [0.68 - 1.11]	**0.69 [0.54 - 0.89]**	**0.48 [0.37 – 0.61]**
Surinamese	6,171	**3.09 [2.66 – 3.60]**	**3.06 [2.63 - 3.57]**	**2.79 [2.40 - 3.25]**	**1.99 [1.70 – 2.33]**
Antillean, Aruban	2,687	**4.54 [3.76 – 5.49]**	**4.53 [3.75 - 5.47]**	**3.59 [2.97 - 4.35]**	**2.48 [2.04 – 3.01]**
Non-western, other	11,950	**1.70 [1.47 – 1.97]**	**1.69 [1.47 - 1.96]**	**1.38 [1.19 - 1.60]**	1.05 [0.91 – 1.22]
Western, other	24,057	**1.53 [1.37 – 1.71]**	**1.52 [1.36 - 1.70]**	**1.50 [1.34 - 1.67]**	**1.24 [1.11 – 1.39]**
Generation of EM					
Native Dutch	221,184	Reference	Reference	Reference	Reference
1^st^ generation	34,591	**1.52 [1.38 – 1.67]**	**1.50 [1.36 - 1.65]**	**1.46 [1.32 - 1.61]**	1.09 [0.98 – 1.20]
2^nd^ generation	23,983	**2.21 [2.00 – 2.43]**	**2.20 [2.00 - 2.43]**	**1.69 [1.53 - 1.86]**	**1.30 [1.18 – 1.44]**

^a^ OR adjusted for: gender

^b^ OR adjusted for: gender, age

^c^ OR adjusted for: gender, age, degree of urbanization

In bold: OR is statistically significant (p < 0.05)

OR: Odds Ratio; aOR: adjusted Odds Ratio; EM: ethnic minorities; CI: confidence interval

## Discussion

At general practices in the Netherlands, EM consult the GP more often for STI care than native Dutch, except for Turkish EM. A lower age and a higher degree of urbanization explained the higher consultation rate among Moroccan and non-Western EM, but not among Antillean/Aruban, Surinamese and other Western EM. In addition, the percentage of episodes that was STI-related was higher for EM (except for Turkish EM) than for native Dutch.

### Strengths and limitations

A strength of our study is that we were able to match the majority of the patients registered in a large nationally representative general practice database with the population register, which made it possible to determine the patients’ ethnicity, since this is not registered in the general practice data [23].

A limitation was that 33 % of the patients registered in the NIVEL-PCD could not be identified in the population registry, which could have led to selection bias. However, the distribution of EM in the study population was comparable with the total Dutch population [29]. We have no reason to assume that possible selection bias is related to STI-related episodes.

Furthermore, episodes, like vaginitis or vaginal discharge, in which an STI test was performed but with a “non-STI” final diagnosis, do not appear as an STI-related episode in our study, leading to an underestimation of all episodes involving an STI test and to a relatively high proportion of STI-related episodes where an STI was diagnosed. This makes a comparison with the STI positivity rate, for example reported by STI clinics [2], impossible.

Finally, since we did not have information on sexual risk behavior and underlying need for an STI consultation, we could not draw any conclusions on the adequacy of STI care by GPs.

### Discussion of findings

Our findings differ from earlier findings from Trienekens et al. who suggested that EM consult the GP less frequently for STI care than native Dutch [20]. They compared the proportion of EM among people who consulted the GP for STI care (16 %, based on self-defined ethnicity) with the proportion of EM in the overall Dutch population (20 %, based on (parental) country of birth). Since self-defined ethnicity often leads to an underestimation of EM [11], this could explain the difference with our results (in our data 29 % of STI-related episodes was among EM). Van Bergen et al. showed that EM consult their GP more often after experiencing STI-related symptoms than native Dutch, which is in line with our results [17].

Comparing our data to data from STI centers, it seems that Turkish, Moroccan and Antillean/Aruban EM may prefer to consult the GP to the STI center for STI care. In 2011, 1.3 % of the visitors at the STI center was Turkish compared with 2.0 % of the patients with an STI-related episode at the GP. For Moroccan EM these percentages were 1.8 % at the STI center and 3.3 % at the GP, and for Antillean/Aruban EM 2.8 % and 3.7 % respectively [30]. Comparable data from other countries is limited since the regulation of STI care differs widely between countries. In the UK, the ethnicity of women diagnosed with chlamydia in primary care did not differ from those diagnosed in genitourinary medicine clinics [31]. In the US, men attending a public STI clinic were more often black compared to men attending a non-public clinic [32].

Especially Antillean/Aruban and Surinamese EM consulted the GP relatively often for STI care compared with native Dutch. This is in line with previous Dutch research showing a high proportion of these ethnic groups with a previous/recent STI/HIV test [16, 33]. Also in the UK, Black Caribbeans reported more frequently a genitourinary medicine clinic visit and a previous HIV test than white ethnic groups [5].

It seems that Antillean/Aruban and Surinamese EM find their way to STI care, while their STI prevalence remains high. The odds of being diagnosed with a single STI at the STI center was about 1.5 times higher among Antillean/Aruban or Surinamese EM than among native Dutch, and the odds of a co-infection was 3.6-5.9 times higher [16]. In a Dutch screening program, the odds of being diagnosed with chlamydia was 3.0-4.3 times higher among Antillean/Aruban or Surinamese EM than among native Dutch [11]. In our study, the odds of having an STI-related episode at the GP was 2.0-2.5 times higher among Antillean/Aruban or Surinamese EM than among native Dutch. Although the higher STI consultation rate at the GP among Antillean/Aruban and Surinamese EM is reassuring, we are not certain that this covers their higher need.

In our study, there was no difference in the STI consultation rate between Moroccan EM and native Dutch (after adjustment for gender, age and degree of urbanization), while Turkish EM had a lower consultation rate. For Moroccan and Turkish EM it is debatable whether they are at higher STI risk than native Dutch. At the STI center, Moroccan or Turkish women had a 1.2 higher odds of being diagnosed with a single STI than native Dutch women, and a 2.2 higher odds of having a co-infection, while there was no increased risk among Moroccan or Turkish men [16]. The chlamydia positivity rate in a screening program was higher for Moroccan EM than for native Dutch (OR: 1.9) while there was no difference between Turkish EM and native Dutch (OR: 1.0) [11]. It is uncertain whether this is a reflection of the general Moroccan and Turkish population, since only a selective group will come forward to get tested.

In contrast to other ethnic groups, Moroccan and Turkish women consulted the GP less often for STI care than men. This may be culturally related since Islamic women are expected to keep their virginity until marriage [34]. A Belgium study showed that Moroccan women are less concerned about safe sex, as they do not have sex before marriage. However, premarital sex seems to be acceptable for Moroccan men [35]. Also in the Netherlands young Islamic women find that remaining a virgin until marriage is stricter for women than for men [36]. Indeed, Moroccan and Turkish women seem to have lower sexual risk behavior (less sexual experience at young age and less sexual partners) than native Dutch women, although their condom use is lower. Moroccan and Turkish men seem to have higher sexual risk behavior than native Dutch men (more sexual experience at young age, more sexual partners, more often commercial sex) [18, 33, 35, 37].

Currently, the ethnic background of patients is often not registered in general practices in the Netherlands. Nevertheless, ethnic background is an important determinant for many diseases, including STIs. Although some are concerned it may be perceived as stigmatizing, including ethnicity in patient registers and surveillance data will give more insight into the influence of ethnicity on patients’ health and use of primary health care and would enable a more focused approach regarding service provision, education and further research in the care of this important patient group within primary care [22].

## Conclusion

In conclusion, EM consult their GP more often for STIs than native Dutch, but it is unknown whether this covers the need of EM groups at higher STI risk. The GP has an important role in STI care, especially in EM groups; it is therefore imperative that GPs use the opportunity of an STI-consultation (or other chances) to offer the complete STI-test package (including HIV), following the rules from the recently renewed guideline for STI consultations at the general practice [38].

## Additional files

Additional file 1:
**Characteristics of the study population per year (2002 to 2011).**
Additional file 2:
**Characteristics of the study population in 2011, by ethnicity and generation of ethnic minorities (EM).**

## Abbreviations

aOR: Adjusted Odds Ratio
ATC: Anatomical Therapeutic Chemical Classification System
CI: Confidence interval
EM: Ethnic minorities
GP: General practitioners
ICPC: International Classification of Primary Care
NIVEL: Netherlands Institute for Health Sciences Research
NIVEL-PCD: NIVEL Primary Care Database
OR: Odds Ratio
PY: Patient year
STI: Sexually transmitted infection
