# Supplementary material for: Comparison of STI-related consultations among ethnic groups in the Netherlands: an epidemiologic study using electronic records from general practices
Source: BMC Fam Pract. 2015 Jun 18;16:70. doi: 10.1186/s12875-015-0281-2 (PMC4470336; doi:10.1186/s12875-015-0281-2)
Supplement: Additional file 1: — Characteristics of the study population per year (2002 to 2011). [file 12875_2015_281_MOESM1_ESM.docx]

# Additional file 1

**Characteristics of the study population per year (2002 to 2011).**

|  | **2002**  **(%)** | **2003**  **(%)** | **2004**  **(%)** | **2005**  **(%)** | **2006**  **(%)** | **2007**  **(%)** | **2008**  **(%)** | **2009**  **(%)** | **2010**  **(%)** | **2011**  **(%)** |
| --- | --- | --- | --- | --- | --- | --- | --- | --- | --- | --- |
| **Total (N)** | 262454 | 245428 | 207478 | 195152 | 242121 | 284700 | 285387 | 269033 | 292088 | 279758 |
| **Gender**­ |  |  |  |  |  |  |  |  |  |  |
| Male | 50.0 | 50.0 | 50.2 | 50.2 | 49.9 | 49.6 | 49.5 | 49.6 | 49.6 | 49.9 |
| Female | 50.0 | 50.0 | 49.8 | 49.8 | 50.1 | 50.4 | 50.5 | 50.4 | 50.4 | 50.1 |
| **Ethnicity** |  |  |  |  |  |  |  |  |  |  |
| Native Dutch | 83.2 | 83.2 | 82.9 | 82.6 | 80.9 | 79.6 | 79.7 | 80.1 | 78.5 | 79.1 |
| Moroccan | 1.1 | 1.0 | 1.2 | 1.3 | 1.6 | 1.9 | 1.9 | 2.0 | 2.1 | 2.2 |
| Turkish | 1.5 | 1.3 | 1.4 | 1.5 | 1.8 | 2.3 | 2.3 | 2.3 | 2.7 | 2.7 |
| Surinamese | 1.6 | 1.7 | 1.8 | 1.9 | 2.1 | 2.3 | 2.2 | 2.3 | 2.4 | 2.2 |
| Antillean, Aruban | 0.7 | 0.8 | 0.8 | 0.9 | 1.0 | 1.0 | 1.0 | 1.0 | 1.0 | 1.0 |
| Non-western, other | 2.9 | 3.2 | 3.5 | 3.6 | 4.0 | 4.1 | 4.1 | 3.9 | 4.4 | 4.3 |
| Western, other | 8.9 | 8.8 | 8.4 | 8.4 | 8.6 | 8.8 | 8.8 | 8.5 | 8.8 | 8.6 |
| **Generation of EM (% of EM)** | |  |  |  |  |  |  |  |  |  |
| 1^st^ generation | 58.9 | 58.8 | 59.5 | 59.6 | 60.2 | 60.2 | 59.7 | 57.8 | 59.6 | 59.1 |
| 2^nd^ generation | 41.1 | 41.2 | 40.5 | 40.4 | 39.8 | 39.8 | 40.3 | 42.2 | 40.4 | 40.9 |
| **Age** |  |  |  |  |  |  |  |  |  |  |
| 15-24 years | 18.1 | 18.3 | 18.7 | 18.8 | 18.5 | 18.7 | 19.1 | 18.8 | 19.7 | 19.7 |
| 25-34 years | 24.6 | 23.6 | 22.8 | 22.0 | 21.8 | 21.2 | 20.7 | 19.2 | 19.5 | 19.4 |
| 35-44 years | 25.5 | 25.7 | 25.7 | 25.5 | 26.0 | 25.9 | 25.5 | 25.3 | 23.8 | 23.3 |
| 45-60 years | 31.9 | 32.4 | 32.8 | 33.6 | 33.7 | 34.3 | 34.7 | 36.7 | 37.1 | 37.7 |
| **Degree of urbanization*** | |  |  |  |  |  |  |  |  |  |
| Very high | 16.2 | 16.0 | 15.0 | 14.8 | 18.0 | 21.6 | 21.2 | 19.0 | 21.1 | 19.2 |
| High | 23.1 | 23.3 | 25.4 | 22.6 | 21.0 | 19.8 | 19.2 | 18.9 | 18.7 | 18.9 |
| Moderately high | 16.1 | 18.2 | 18.1 | 18.6 | 16.0 | 15.5 | 15.6 | 17.1 | 15.8 | 14.7 |
| Low | 23.9 | 21.1 | 21.1 | 23.0 | 21.7 | 20.9 | 21.0 | 21.2 | 21.4 | 21.5 |
| Very low | 19.9 | 21.3 | 20.2 | 20.8 | 23.1 | 21.9 | 22.9 | 23.7 | 23.1 | 25.7 |
| * Based on population density per postal code area, missing for some patients. | | | | | | | | | | |
| EM = ethnic minorities | | | | | | | | | | |
