# Supplementary material for: Comparison of STI-related consultations among ethnic groups in the Netherlands: an epidemiologic study using electronic records from general practices
Source: BMC Fam Pract. 2015 Jun 18;16:70. doi: 10.1186/s12875-015-0281-2 (PMC4470336; doi:10.1186/s12875-015-0281-2)
Supplement: Additional file 2: — Characteristics of the study population in 2011, by ethnicity and generation of ethnic minorities (EM). [file 12875_2015_281_MOESM2_ESM.docx]

# Additional file 2

**Characteristics of the study population in 2011, by ethnicity and generation of ethnic minorities (EM).**

|  | | **Ethnicity** | | | | | | |  | **Generation of EM** | |
| --- | --- | --- | --- | --- | --- | --- | --- | --- | --- | --- | --- |
|  | | Native Dutch | Moroccan | Turkish | Surinamese | Antillean, Aruban | Non-western, other | Western, other |  | 1^st^ generation | 2^nd^ generation |
| **Total (N)** | | 221184 | 6220 | 7489 | 6171 | 2687 | 11950 | 24057 |  | 34591 | 23983 |
| **Gender**­ (%) | |  |  |  |  |  |  |  |  |  |  |
| Male | | 50.5 | 47.8 | 50.3 | 46.1 | 48.4 | 48.5 | 46.5 |  | 46.1 | 49.8 |
| Female | | 49.5 | 52.2 | 49.7 | 53.9 | 51.6 | 51.5 | 53.5 |  | 53.9 | 50.2 |
| **Age (%)** | |  |  |  |  |  |  |  |  |  |  |
| 15-24 years | | 19.3 | 30.2 | 26.1 | 22.7 | 29.0 | 23.2 | 15.0 |  | 11.0 | 35.8 |
| 25-34 years | | 17.9 | 29.2 | 26.8 | 21.9 | 26.7 | 28.7 | 22.3 |  | 25.6 | 24.4 |
| 35-44 years | | 23.0 | 24.9 | 24.9 | 22.4 | 20.9 | 23.4 | 25.5 |  | 29.4 | 17.2 |
| 45-60 years | | 39.9 | 15.7 | 22.2 | 33.0 | 23.4 | 24.6 | 37.2 |  | 34.0 | 22.7 |
| **Degree of urbanization* (%)** | | | |  |  |  |  |  |  |  |  |
| Very high | 13.9 | | 48.8 | 52.8 | 53.1 | 52.7 | 38.9 | 27.6 |  | 43.3 | 33.3 |
| High | 17.6 | | 20.7 | 26.4 | 21.1 | 24.2 | 26.2 | 23.3 |  | 23.9 | 23.7 |
| Moderately high | 15.4 | | 7.7 | 10.3 | 7.1 | 11.0 | 12.7 | 15.2 |  | 11.4 | 13.4 |
| Low | 22.9 | | 19.9 | 8.9 | 13.9 | 8.0 | 14.3 | 19.6 |  | 14.2 | 18.8 |
| Very low | 30.3 | | 2.9 | 1.5 | 4.8 | 4.0 | 7.8 | 14.2 |  | 7.2 | 10.7 |
| * Based on population density per postal code area, missing for some patients. | | | | | | | | | | | |
